# Coagulopathy and Hospital Outcomes in Patients With Spontaneous Bacterial Peritonitis: A Call for Action to Improve Care of Inpatients

**DOI:** 10.7759/cureus.8926

**Published:** 2020-06-30

**Authors:** Gayathri Gurumurthy, Anusha Gaddam, Viralkumar Patel, Rikinkumar S Patel

**Affiliations:** 1 Internal Medicine, Shadan Institute of Medical Sciences, Hyderabad, IND; 2 Internal Medicine, Chalmeda Anand Rao Institute of Medical Sciences, Karimnagar, IND; 3 Internal Medicine, Blake Medical Center, Bradenton, USA; 4 Psychiatry, Griffin Memorial Hospital, Norman, USA

**Keywords:** bacterial peritonitis, mortality, hospital outcomes, coagulopathy, coagulation disorders, national inpatient sample

## Abstract

Objectives

To assess the risk of in-hospital mortality in spontaneous bacterial peritonitis (SBP) with coagulopathy, and to understand the impact of comorbid coagulopathy on length of stay (LOS) and total charges for SBP inpatients.

Methods

We included adult patients (age, 18-50 years) with a principal diagnosis of SBP using the Nationwide Inpatient Sample (NIS, 2012 to 2014). These patients were further subgrouped by comorbid coagulopathy. The independent sample t-test was used to measure the mean difference in LOS and total charges between subgroups. The logistic regression model was used to measure the odds ratio (OR) of association of coagulopathy and in-hospital mortality after adjusting for demographic confounders and other comorbid risk factors.

Results

SBP with comorbid coagulopathy was prevalent in males (68.7%) and white (58.1%). When compared with the non-coagulopathy cohort, males had 1.6 times (95% CI 1.46-1.84), and hispanics had 1.4 times (95% CI 1.19-1.58) high odds for coagulopathy. In-hospital mortality was statistically significant in SBP inpatients with coagulopathy (6.5% vs. 2.8% in non-coagulopathy), and with two times higher odds of association (95% CI 1.47-2.51) compared with non-coagulopathy cohort. SBP inpatients with comorbid coagulopathy had a statistically significantly higher LOS by 1.1 days and higher total charges by $14,123 per hospitalization compared with the non-coagulopathy cohort.

Conclusions

Coagulopathy is a significant risk factor that increases the risk of in-hospital mortality in SBP inpatients by 92%. Comorbid coagulopathy is also associated with extended LOS and higher hospitalization costs, thereby increasing the healthcare burden. Clinicians need to effectively manage coagulopathy in SBP patients to improve patient outcomes and reduce the healthcare burden with better health-related quality of life.

## Introduction

Spontaneous bacterial peritonitis (SBP) is the most common bacterial infection of the ascitic fluid occurring in patients with cirrhosis. SBP is prevalent in 11%-14% of patients hospitalized for cirrhosis and ascites. SBP-related in-hospital mortality has a strong association with certain medical comorbidities, including hepatic encephalopathy, coagulopathy, variceal hemorrhage, sepsis, pneumonia, acute kidney injury [1].

Advanced liver disease is characterized by alterations in hemostasis and dysfunction of the hepatic reticuloendothelial system which is correlated to the risk of acquiring SBP in patients with cirrhosis. Patients with chronic liver disease have high levels of endotoxins present in the portal and systemic circulation, and these endotoxin levels are further exacerbated by bacterial infections. Endotoxins through the release of endothelins, nitric oxide, and cyclooxygenase products cause increased portal pressure, inhibition of platelet aggregation, and further impairment of primary hemostasis eventually leading to variceal hemorrhage and other life-threatening complications of cirrhosis [2].

Based on past studies, cirrhotic patients with active infection have shown a hypercoagulable state with an increased risk of bleeding. Bacterial infections play a part in causing this hypercoagulable state through the production of heparin-like substances by inhibiting the activated clotting factor Xa [3,4]. Disturbances in fibrinolysis, impaired protein synthesis, endothelial dysfunctions, and decreased platelet function all play an important role in contributing towards coagulopathy in advanced liver disease and increase the risk of bleeding and thromboembolic events [5]. Venous thromboembolism in the form of pulmonary embolism, deep venous thrombosis, or portal vein thrombosis all constitutes a significant cause for morbidity and mortality due to coagulopathy in patients with SBP [6]. However, upper gastrointestinal bleeding is a vital cause for mortality and it constitutes a major site for bleeding in patients with advanced liver disease [6].

To our knowledge, the existing studies are broad based and focused on multiple risk factors associated with SBP. In our study, we aim to determine the demographic predictors and risk of chronic comorbidities in SBP with comorbid coagulopathy. Next, we want to assess the risk of in-hospital mortality due to comorbid coagulopathy after adjusting the regression model for chronic comorbidities, and lastly to understand the impact of comorbid coagulopathy on length of stay (LOS) and total charges for SBP hospitalization.

## Materials and methods

Data source

We conducted a cross-sectional data analysis using the Nationwide Inpatient Sample (NIS) from 2012 to 2014. The NIS provides patient information from about 4,400 hospitals across 44 states in the United States (US). Diagnostic information in the NIS is distinguished using the International Classification of Diseases, Ninth Edition (ICD-9) codes. To protect the patient's identity and health information, the NIS was de-identified and so we do not require approval from the institutional review board [7].

Inclusion criteria and outcome variables

We included 6,530 adult patients (age, 18-50 years) with a principal diagnosis of SBP using ICD-9 code 567.23, further subgrouped by comorbid coagulopathy (N = 2,555).

The demographic variables studied in this analysis were age, sex, and race. Chronic comorbidities of chronic alcoholic liver disease (CALD), diabetes, hypertension, congestive cardiac failure (CCF), and renal failure were identified using ICD-9 diagnosis codes. In-hospital mortality in the NIS is reported as all-cause, and other hospital outcomes included were LOS and total charges [8].

Statistical analysis

We used descriptive statistics to discern the differences in demographics and comorbidities, and in-hospital mortality in SBP inpatients by comorbid coagulopathy. The logistic regression model was used to evaluate the demographic predictors and association of comorbidities in SBP inpatients with coagulopathy, and its impact on odds ratio (OR) association with in-hospital mortality. The independent sample t-test with equality measures was used to measure the differences between cohorts for LOS and total charges. A P-value of <0.01 was used to assess the statistical significance of data analyses conducted on the Statistical Package for the Social Sciences (SPSS) version 26 (IBM Corporation, Armonk, NY).

## Results

We analyzed a sample of 6,530 SBP inpatients with 39.1% having comorbid coagulopathy. Approximately 84.3% of the SBP inpatients with coagulopathy were middle-age adults 36 to 50 years having 1.3 times higher odds (95% CI 1.18-1.57) for coagulopathy compared with 15.7% of the young adults. A higher proportion of SBP inpatients with coagulopathy were male (68.7%) and white (58.1%). When compared with the non-coagulopathy cohort, males had 1.6 times (95% CI 1.46-1.84), and hispanics had 1.4 times (95% CI 1.19-1.58) high odds for coagulopathy than their counterparts.

The most prevalent comorbidities seen in SBP inpatients with coagulopathy were CALD (57.1%), hypertension (27%), and diabetes (16.6%). Patients with coagulopathy had two times higher odds for CALD (95% CI 1.81-2.27) compared with the non-coagulopathy cohort. A higher proportion of inpatient deaths were seen in SBP inpatients with coagulopathy (6.5% vs. 2.8%), and with two times higher odds of association (95% CI 1.47-2.51) compared with the non-coagulopathy cohort (Table 1).

**Table 1:** Demographic predictors and association of comorbidities with in-hospital mortality

Variable	Coagulopathy (%)		Logistic regression model		
	(-)	(+)	Odds ratio	95% confidence interval	P-value
Total N	3,975	2,555	-	-	-
Age at admission					
18–35 years	22.3	15.7	Reference		
36–50 years	77.7	84.3	1.36	1.18–1.57	<0.001
Sex					
Male	53.8	68.7	1.64	1.46–1.84	<0.001
Female	46.2	31.3	Reference		
Race					
White	58.5	58.1	Reference		
Black	16.4	9.2	1.23	1.02–1.49	0.030
Hispanic	17.4	23.1	1.37	1.19–1.58	<0.001
Other	7.7	9.6	1.32	1.09–1.61	0.006
Comorbidities					
No comorbidities	-	-	Reference		
Chronic alcoholic liver disease	35.0	57.1	2.02	1.81–2.27	<0.001
Hypertension	39.5	27.0	0.71	0.62–0.81	<0.001
Diabetes	12.2	16.6	1.61	1.37–1.88	<0.001
Congestive heart failure	8.1	2.3	0.35	0.26–0.48	<0.001
Renal failure	27.5	13.1	0.61	0.52–0.72	<0.001
In-hospital mortality					
No deaths	97.2	93.5	Reference		
Deaths	2.8	6.5	1.92	1.47–2.51	<0.001

The mean LOS (7.3 days vs. 6.2 days) and total charges ($61,626 vs. $47,503) for patients with coagulopathy were higher compared with the non-coagulopathy cohort (P < 0.001). SBP inpatients with comorbid coagulopathy had a significantly higher LOS by 1.13 days and higher total charges by $14,123 per hospitalization (Table 2).

**Table 2:** Impact of comorbid coagulopathy on hospitalization stay and total charges SD: standard deviation; $: United States dollars

Variable	Coagulopathy	
	(-)	(+)
Length of stay, in days		
Mean ±SD	6.15 ±6.39	7.29 ±7.30
Mean difference	-1.13	
95% confidence interval	-1.47 to -0.79	
P-value	<0.001	
Total charges, in $		
Mean ±SD	47,503 ±70,988	61,626 ±83,475
Mean difference	-14123	
95% confidence interval	-17,928 to -10,318	
P-value	<0.001	

## Discussion

Comorbid coagulopathy was prevalent in 39% of SBP inpatients and about four-fifths of these patients were middle-aged adults. Aging is an adverse prognostic factor in liver diseases as it impairs immunity and increases susceptibility to infections [9]. As per a study by Niu et al., the mean age of SBP inpatients was 56.2 years and the mean age of the patients who died was 58.2 years [1]. Furthermore, our study identified a higher prevalence of coagulopathy in men with SBP, and this could be due to increased consumption of alcohol among the male population making them more susceptible to CALD. Also, as these patients with SBP have pre-existing liver cirrhosis or other related problems, they are prone to hypercoagulability. Varying levels of alcohol intake seem to have a range of effects on coagulation, such as lowered platelet count, lowered fibrinogen levels, and other prothrombotic events like an increase in factor VII and anti-fibrinolytic activity [10]. Also, an increase in alcohol intake reduces the plasma fibrinogen concentration by 10%, platelet count by 3%, and an increase in factor VII by 7% [11]. Additionally, the study by Niu et al. had a similar sex distribution wherein the in-hospital mortality was seen in 63.6% males and 36.4% females, which concurs with our findings [1].

About one-third of patients with chronic hepatitis and liver diseases have alcohol use problems, including abuse and dependence [12]. CALD leads to decreased synthesis of clotting and inhibitor factors, decreased clearance of activated factors, quantitative and qualitative platelet defects, hyperfibrinolysis, and accelerated intravascular coagulation [13]. Our study shows a higher prevalence in SBP in patients with coagulopathy having comorbid CALD (risk increased by two times), compared with other risk factors, such as hypertension and diabetes. A recent study found that alcohol consumption has a negative effect on the liver affecting the synthesis of coagulation factors such as factors II, V, VII, and X, and fibrinogen, and it impacts the hemostasis and fibrinolytic system [14,15]. An inpatient study by Bhandari et al. found that CALD is an independent factor that increases the risk of in-hospital mortality in SBP patients by 48% [16].

The prevalence of in-hospital mortality in SBP inpatients with coagulopathy was 6.5%, higher than other inpatients (2.8%). This rate is lower than a recent study conducted by Niu et al., which had a higher mortality rate of 28.3% due to SBP related coagulopathy [1]. This study was conducted on a larger population (N = 170,430) of SBP patients with multiple comorbidities including coagulopathy. Our study found that SBP inpatients with coagulopathy had two times (or 92%) higher odds of in-hospital mortality compared with the non-coagulopathy cohort in adjusted regression analysis. Chronic liver disease commonly presents with low platelet count due to sequestration of platelet in the spleen, and increased platelet breakdown and its decreased production. Infections and sepsis activate platelets bound to the endothelium which further increases their destruction. This eventually reduces the platelet count, leading to massive bleeding [17]. Apart from bleeding, venous thromboembolism is another common cause of increased mortality and morbidity among hospitalized patients [18].

Cirrhotic patients are traditionally at a high risk of bleeding due to higher prothrombin time or international normalized ratio and lower platelet counts. Infections like SBP further reduce the platelet count, resulting in life-threatening complications such as severe bleeding from esophageal varices or other sites, leading to extended intensive care unit (ICU) stays and increased in-hospital mortality [19]. These patients have many other complications such as encephalopathy, infection, and acute kidney injury that can prolong their stay in the hospital. This could be the possible reason for higher inpatient total charges and longer LOS in SBP inpatients with coagulopathy compared with those without coagulopathy in our study.

Few limitations of our study include underreporting of coagulopathy as being an administrative data based on ICD-9 codes with inconsistency in diagnostic codes applied during patient billing. Also, in-hospital mortality in this study is all-cause which does not prove a causal relationship between mortality and comorbidities in SBP patients including coagulopathy. Some of the strengths of our study include nationwide data analysis covering 44 states across the US with external validity to the American population, and strong methodology including demographic adjusted regression model to evaluate the risk of association with mortality in SBP inpatients.

## Conclusions

Coagulopathy was predominantly prevalent in SBP patients, and middle-age black and/or hispanic men have a higher risk of a co-diagnosis of coagulopathy with SBP. Comorbid coagulopathy was a significant risk factor that increases the odds of in-hospital mortality by 92%. It was also associated with extended LOS and higher hospitalization costs thereby increasing healthcare burden. Clinicians need to effectively manage coagulopathy in SBP patients to improve patient outcomes and reduce the healthcare burden with better health-related quality of life.
